# Subculture of Germ Cell-Derived Colonies with GATA4-Positive Feeder Cells from Neonatal Pig Testes

**DOI:** 10.1155/2016/6029271

**Published:** 2016-01-06

**Authors:** Kyung Hoon Lee, Won Young Lee, Jin Hoi Kim, Chan Kyu Park, Jeong Tae Do, Jae Hwan Kim, Young Suk Choi, Nam Hyung Kim, Hyuk Song

**Affiliations:** ^1^Department of Animal Biotechnology, College of Animal Bioscience & Technology, Konkuk University, Seoul 143-701, Republic of Korea; ^2^Department of Food Bioscience, College of Biomedical & Health Science, Konkuk University, Chungju 380-701, Republic of Korea; ^3^Department of Biomedical Science, College of Life Science, CHA University, Seongnam 463-836, Republic of Korea; ^4^Department of Animal Science, College of Agriculture, Chungbuk National University, Cheongju 361-763, Republic of Korea

## Abstract

Enrichment of spermatogonial stem cells is important for studying their self-renewal and differentiation. Although germ cell-derived colonies (GDCs) have been successfully cultured from neonatal pig testicular cells under 31°C conditions, the short period of* in vitro* maintenance (<2 months) limited their application to further investigations. To develop a culture method that allows for* in vitro* maintenance of GDCs for long periods, we subcultured the GDCs with freshly prepared somatic cells from neonatal pig testes as feeder cells. The subcultured GDCs were maintained up to passage 13 with the fresh feeder cells (FFCs) and then frozen. Eight months later, the frozen GDCs could again form the colonies on FFCs as shown in passages 1 to 13. Immunocytochemistry data revealed that the FFCs expressed GATA-binding protein 4 (GATA4), which is also detected in the cells of neonatal testes and total testicular cells, and that the expression of GATA4 was decreased in used old feeder cells. The subcultured GDCs in each passage had germ and stem cell characteristics, and flow cytometric analyses revealed that ~60% of these cells were GFR*α*-1 positive. In conclusion, neonatal pig testes-derived GDCs can be maintained for long periods with GATA4-expressing testicular somatic cells.

## 1. Introduction

Spermatogonial stem cells (SSCs) are the first precursors to spermatogenic cells (spermatocytes, spermatids, and spermatozoa) and are essential for continued spermatogenesis throughout the life span [1]. Owing to their small population, long-term culture-mediated enrichment of SSCs is important for investigating their self-renewal and differentiation. To date, long-term culture of SSCs has only been reported in rodents [2]. A 5-month culture of mice SSCs has been reported, where mouse embryonic fibroblasts (MEFs) were used as feeder cells [3]. Hamster SSCs cultured with mitomycin-C-treated MEFs for 3 passages have been cultured for 1 year without feeder cells [4]. Additionally, mice SSCs cultured with SIM mouse embryo-derived thioguanine and ouabain-resistant (STO) cell feeders have been shown to proliferate for 7 months [5]. In humans, stage-specific embryonic antigen-4-positive SSCs have been cultured for 4 months without feeder cells [4]. Furthermore, transplantation of mice SSCs results in the production of sperms in recipient mice. In contrast to rodents, long-term culture of SSCs is difficult in domestic animals because of the unavailability of feeder cells (like MEFs or STO cells) for subculture, although short-term culture (<2 months) has been reported [6]. In our previous study, we reported that male germ cell-derived colonies (GDCs) effectively formed in 10 days at 31°C and that the transplanted GDCs colonized recipient testes at 8 weeks after transplantation [7]. In this study, we demonstrate a method for the subculture and enrichment of porcine GDCs using feeder cells isolated from neonatal pig testes.

## 2. Materials and Methods

### 2.1. Sample Preparation

Ten testes from castrated Yorkshire- and Landrace-crossed, 5-day-old, hybrid piglets were obtained from the Samwoo Livestock (Yangpyeong, Korea). Tunicae albugineae of the testes were removed for immunocytochemistry and cell culture preparations. The study protocol and standard operating procedures were reviewed and approved by the Institutional Animal Care and Use Committee of Konkuk University (IACUC approval number: KU13035).

### 2.2. Feeder Cell Preparation

Feeder cells were prepared according to a previously reported protocol with some modifications [8]. Five volumes (v/w) of enzyme A (0.5 mg/mL collagenase, 0.01 mg/mL DNase I, 0.1 mg/mL soybean trypsin inhibitor, and 0.1 mg/mL hyaluronidase) were added to the decapsulated testes, and the testes were incubated for 10 min at 37°C. The testes were then washed with phosphate-buffered saline (PBS). Next, 5 volumes (v/w, original testes weights) of enzyme B (5 mg/mL collagenase, 0.01 mg/mL DNase I, and 0.1 mg/mL soybean trypsin inhibitor) were added, and the testes were incubated for a further 10 min at 37°C. The enzyme-treated testes were then washed with PBS and meshed using a 40 *μ*m nylon mesh. Red blood cells were eliminated using a lysis buffer (Sigma-Aldrich, St. Louis, MO, USA). Two Percoll densities, 20% and 40% (Sigma-Aldrich), were used to collect feeder cells. The 40% Percoll suspension was prepared by mixing 40% Percoll solution, 10% PBS (v/w), 1% fetal bovine serum (FBS, v/w), 0.5% antibiotics (v/w, 50 U/mL and 50 *μ*g/mL penicillin and streptomycin, resp.), and 48.5% ultrapure water (v/w) in a 15 mL conical tube. The 20% suspension was prepared by diluting the isoosmotic 40% Percoll suspension with PBS supplemented with 1% FBS (v/w) and antibiotics (as above). The cell suspension (2 mL of 5 × 10^6^ cells/mL) was loaded on top of the gradient and centrifuged at 600 ×g for 10 min at 4°C. The cells at top layer (40% Percoll layer) were collected and used as feeder cells. They were analyzed by immunocytochemistry.

### 2.3. Cell Culture for GDCs and Subculture with Fresh Feeder Cells (FFCs)

Total testicular cell (TTC) culture was performed to obtain GDCs. The protocol for single cell preparation was the same as that used for the feeder cells. The isolated cells were seeded onto 0.2% (w/v) gelatin-coated 12-well plates and incubated at 31°C in a 5% CO_2_ atmosphere. The StemPro-34 medium (Gibco, Carlsbad, CA, USA) was used for all processes, including derivation and culture of porcine GDCs. The medium was supplemented with the following: insulin-transferrin-selenium (ITS, 25 *μ*g/mL, 100 *μ*g/mL, or 30 nM), 6 mg/mL glucose, 2 mM L-glutamine, 1% NEAA solution, 1% vitamin solution, 100 units/mL penicillin/streptomycin, 1 mM sodium pyruvate, 0.1 mM vitamin C, 1 *μ*g/mL lactic acid, 30 ng/mL estradiol, 60 ng/mL progesterone, 0.2% bovine serum albumin (BSA), 1% knockout serum replacement, 20 ng/mL mEGF, 10 ng/mL bFGF, 10 ng/mL GDNF, and 10^3^ U/mL leukemia inhibitory factor. The GDCs were collected on days 10 to 12. They were suspended as single cells using 0.25% trypsin-EDTA and then moved to new gelatin-coated tissue culture plates with feeder cells; in total, 2 × 10^4^ GDCs and 1.8 × 10^5^ feeder cells were added to each well. To subculture the GDCs, two kinds of feeder cell were used: old feeder cells (OFCs)—those attached to the wells in passages 0 and 1 after colony collection on days 10 to 12—and FFCs—those isolated from the top of the 40% Percoll layer during density separation of the TTCs. The cells from GDCs in passage 13 were frozen with 10% dimethyl sulfoxide (DMSO) and cultured with FFCs 8 months later.

### 2.4. Cell Labeling

To examine whether GDCs can be maintained in subculture, cells derived from them were labeled with PKH26 red fluorescent dye (Sigma-Aldrich) according to the manufacturer's instructions. The cells were isolated from the colonies in passages 1 and 13 by treatment with 0.25% trypsin for 10 min at 37°C. The cells from the GDCs at passages 1 and 10 were labeled with PKH26 and then used as germ cells in passages 2 and 11. They were then observed with an excitation filter of 450–560 nm under a fluorescence microscope (Nikon, Tokyo, Japan) at a magnification of 400x.

### 2.5. Subculture of Frozen Colony Cells

The GDC cells in passage 13 were frozen in 10% DMSO and the StemPro-34 medium for 8 months. These cells were thawed at 37°C for 1 min, labeled with PKH26 red fluorescent dye (Sigma-Aldrich) (as per manufacturer's instructions), and then cultured with FFCs. They were then observed using an excitation filter of 450–560 nm under a fluorescence microscope (Nikon) at a magnification of 100x.

### 2.6. Immunocytochemistry

Following Percoll gradient separation, cells from the top layer were fixed with 4% paraformaldehyde (PFA, w/v) in PBS and then washed with PBS. Next, they were attached to amino saline-coated slides (Matsunami, Osaka, Japan) for immunocytochemistry. The fixed cells were incubated with the antibody against protein gene product (PGP 9.5) (Santa Cruz Biotechnology, Santa Cruz, CA, USA), raised in rabbits against human PGP 9.5 (1 : 100; AbD Serotec, Raleigh, NC, USA), at 4°C overnight. Following washing with PBS, the cells were further incubated with anti-rabbit Alexa 568 (1 : 500; Invitrogen, Carlsbad, CA, USA) against the PGP 9.5 antibody. The cell nuclei were stained with 4′,6′-diamidino-2-phenylindole (DAPI) staining solution (Vector Laboratories, Burlingame, CA, USA). The same procedure was followed for cells of the colonies in passages 2 and 11, using antibodies against PGP 9.5 and GDNF family receptor alpha-1 (GFR*α*-1) (1 : 50; Santa Cruz Biotechnology). Anti-mouse Alexa 488 (1 : 500; Invitrogen) was used as secondary antibody. Feeder cells attached to the wells at passage 2 were incubated with antibody against GATA-binding protein 4 (GATA4) at 4°C overnight and then washed with PBS. The cells were then incubated with horseradish peroxidase- (HRP-) conjugated secondary antibody (1 : 500; Santa Cruz Biotechnology) for 1 h at room temperature. This was followed by incubation with 3,3′-diaminobenzidine for staining cell nuclei (Vector Laboratories). TTCs that were collected before cell culture were double-stained with antibodies against PGP 9.5 and GATA4 (1 : 50; Santa Cruz Biotechnology). The procedure was the same as that for PGP 9.5 and GFR*α*-1 double staining. Finally, the cells were observed using an excitation filter of 450–560 nm under a fluorescence microscope (Nikon).

### 2.7. Immunohistochemistry

Briefly, 6 *μ*m thick sections of the testis from 5-day-old piglets were deparaffinized with xylene and treated with ethanol. The sections were incubated with a target unmasking fluid (Accurate Chemical & Scientific Corp., Westbury, NY, USA) for 15 min using a microwave oven to retrieve the antigens. The slides were then washed thrice with PBS and blocked with 10% normal goat serum (v/v). For double staining, the slides were incubated with anti-GATA4 (1 : 100; Santa Cruz Biotechnology) and anti-PGP 9.5 antibodies (1 : 100; AbD Serotec, Raleigh, NC, USA) at 4°C overnight and then washed thrice with PBS. Some of the sections were incubated with 1% BSA as negative controls. Next, the sections were incubated with anti-mouse Alexa 488 and anti-rabbit Alexa 568 (both 1 : 500; Invitrogen) against anti-GFR*α*-1 and anti-PGP 9.5 antibodies, respectively, for 1 h at 25°C (room temperature). This was followed by incubation with DAPI (Vector Laboratories). The slides were then washed with PBS and observed using an excitation filter of 450–560 nm under a fluorescence microscope (Nikon) at a magnification of 200x.

### 2.8. Alkaline Phosphatase (AP) Staining

AP staining was performed using a CBA-300 AP staining kit (Cell Biolabs, San Diego, CA, USA) according to the manufacturer's instructions. Briefly, cultured cells including GDCs were fixed in fixation solution, washed thrice with PBS, and incubated with the AP staining solution for 10–20 min. Next, the AP solution was removed and the cells were washed with PBS and observed under a microscope at a magnification of 200x.

### 2.9. RT-PCR

Total RNA from the GDC cells, FFCs, and OFCs was isolated using the RNeasy Mini Kit (Qiagen, Venlo, Netherlands). cDNA templates were prepared from 1000 ng of total RNA using the Maxime RT Premix kit (Intronbio, Seongnam, Korea). The synthesis conditions involved 1 cycle of 60 min at 94°C. cDNA synthesis was inactivated by heating the samples for 5 min at 95°C. Gene-specific primers for beta-2-microtubulin (*B2M*),* PGP 9.5*,* GFRα-1*, promyelocytic leukemia zinc finger (*PLZF*), octamer-binding protein 4 (OCT4), and* NANOG* were used as shown in our previous study [8]. The cycling conditions were as follows: 33 cycles each of 1 min at 94°C, 1 min at 55–60°C, and 2 min at 72°C for all the genes. The PCR products were detected by electrophoresis in a 1.5% agarose gel using Tris-acetate-EDTA buffer.

### 2.10. Indirect Flow Cytometry

The cells collected from colonies in passages 0 and 8 were incubated with anti-GFR*α*-1 antibody (Santa Cruz Biotechnology) for 20 min at 4°C and then washed with FACS buffer (1x PBS, 0.1 *μ*M EDTA, 0.01% sodium azide, and 100 *μ*L FBS). Next, they were incubated with Alexa 488 (Invitrogen) for 20 min at 4°C and analyzed using the FACSCalibur cytometer (BD Biosciences, Franklin Lakes, NJ, USA); the GDC cells treated only with Alexa 488 were used as negative control. All procedures were duplicated using 3 samples from passage 0 and 8 colonies.

## 3. Results

### 3.1. Subculture of GDCs Using FFCs

**Figure 1:**
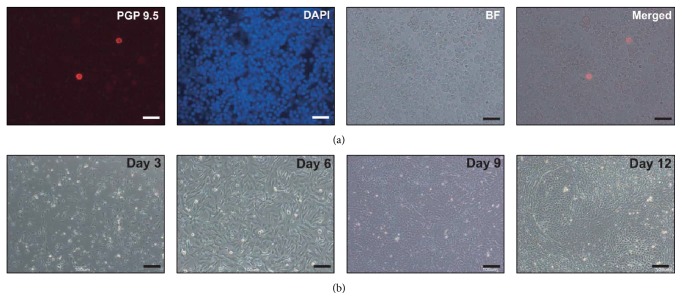
Culture and immunocytochemistry of isolated feeder cells. The isolated feeder cells were stained with PGP 9.5 and DAPI (a) and cultured in the StemPro-34 medium for 12 days (b). Scale bars indicate 30 *μ*m and 100 *μ*m in panels of (a) and (b), respectively.

**Figure 2:**
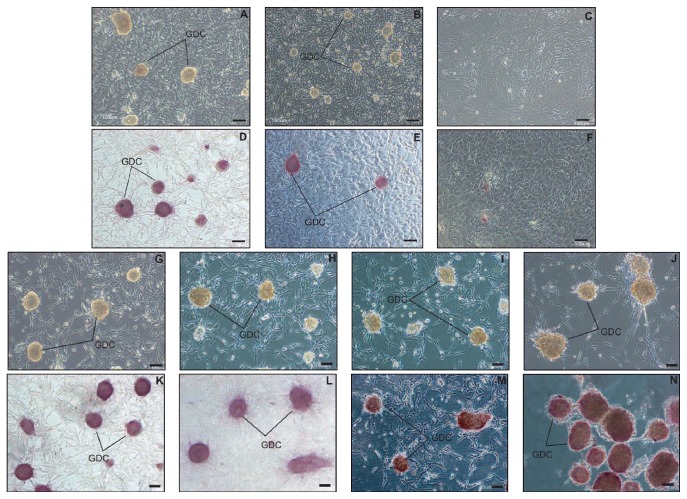
Long-term culture using old and fresh feeder cells. Old feeder cells (OFCs), which were attached to the wells in passages 0 and 1 after colony collection on days 10 to 12, were used for subculture in passages 1 and 2 with the collected colonies at passages 0 and 1, respectively ((A), (B), and (C)). Fresh feeder cells (FFCs), isolated from the top of the 40% Percoll layer during density separation of total testicular cells, were used for subculture of colonies of passages 1 to 7. GDCs in passages 2, 4, 8, and 13 are shown in panels (G), (H), (I), and (J), respectively. Alkaline phosphatase-stained colonies in passages 0, 1, and 2 are shown in panels (D), (E), and (F), respectively. Alkaline phosphatease-stained GDCs in passages 2, 4, 8, and 13 are shown in panels (K), (L), (M), and (N), respectively. Scale bars indicate 100 *μ*m in all panels. GDC: germ cell-derived colony.

In a previous report, we showed that the germ cell-rich layer can be collected by density separation using 20% and 40% Percoll [8]. In this study, we observed only few PGP 9.5-positive cells among those isolated from the top of the 40% Percoll layer upon density separation of the TTCs (Figure 1(a)). A previous study reported the formation of porcine germ cell colonies in StemPro-34 medium at 31°C within days 10 and 12 [7]. In the present study, cells isolated from the top of the 40% Percoll layer were cultured in StemPro-34 medium at 31°C and did not form colonies until day 12, and only the cells attached to the wells showed proliferation (Figure 1(b)). These results indicate that these isolated cells are mostly PGP 9.5 negative. GDCs were observed in passage 0 on day 10 (Figure 2(A)). When cells prepared from GDCs in passage 0 were cultured with OFCs, some colonies formed in passage 1 were smaller than those in passage 0 (Figure 2(B)). Colonies similar to the initial colony in Figure 1(a) were not observed in passage 2 when single cells isolated from GDCs in passage 1 were cultured with OFCs (Figure 2(C)). The colonies in passages 0 and 1 and a few small colonies in passage 2 were positive for AP staining (Figures 2(D)–2(F)). Further, single cells isolated from the GDCs were cultured with FFCs in passage 0. These colonies were formed by subculturing the GDCs. When the cells of colonies in passages 1, 3, 7, and 12 were cultured with FFCs, colony formation was observed in passages 2, 4, 8, and 13 (Figures 2(G)–2(J)); OFCs were used in subculture for all the passages. Similar results were observed in the other passages as well. The colonies were positive for AP staining (Figures 2(K)–2(N)). Between 5.9 × 10^5^ and 1.95 × 10^6^ cells were obtained following colony collection from the 12-well plate (Table 1).

### 3.2. Maintenance and Characterization of GDCs in Subculture

**Figure 3:**
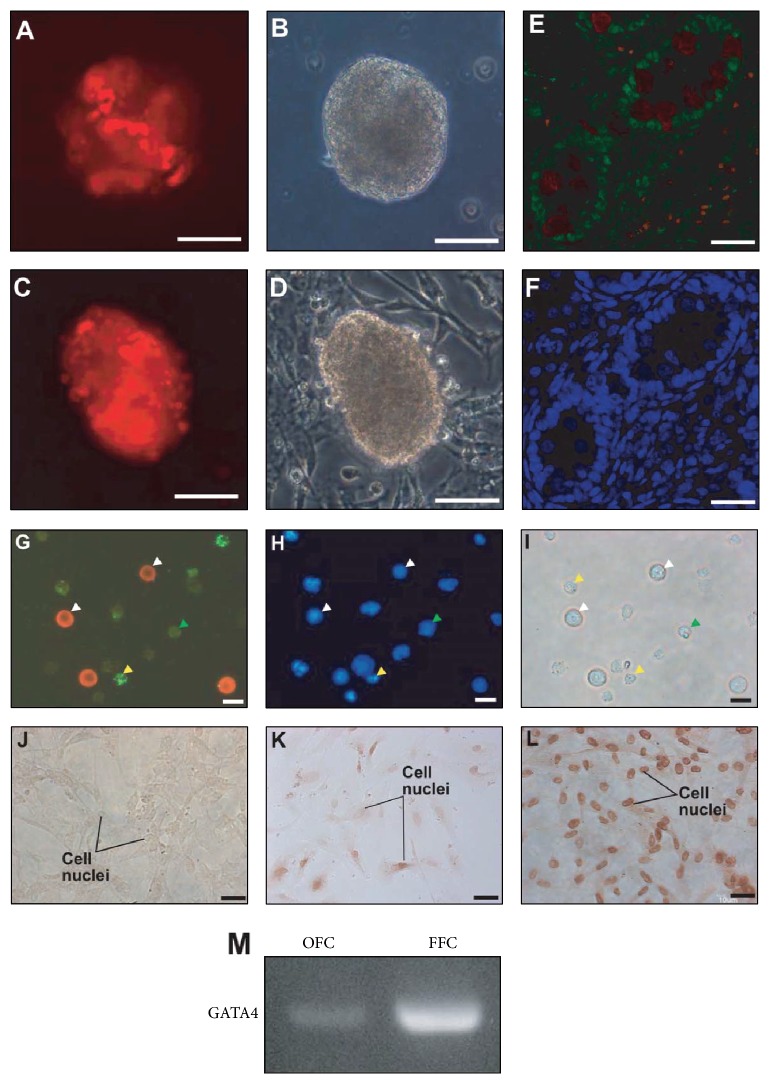
Maintenance of GATA4-positive feeder cells and germ cell-derived colonies (GDCs) in subculture at passages 1 and 10. GDC cells (labeled red) in passages 1 and 10 were maintained in the colonies in passages 2 and 11 ((A) and (C)). Panels (B) and (D) are bright-field images of panels (A) and (C), respectively. GATA4 and PGP 9.5 double staining in neonatal pig testis tissue (E). Panel (F) shows nuclear staining of panel (E). Total testicular cells were stained with antibodies against GATA4 (green) and PGP 9.5 (red) (G). Nuclear staining and bright-field images are shown in panels (H) and (I). Panel (J) shows negative controls on feeder cells attached to the wells. Panels (K) and (L) show GATA4 staining of old feeder cells (OFCs) and fresh feeder cells (FFCs), respectively, in passage 2. White and yellow arrows indicate PGP 9.5- and GATA4-positive cells, respectively; green arrows indicate PGP 9.5- and GATA4-negative cells. Scale bars indicate 50 *μ*m in panels (A)–(F) and 10 *μ*m in panels (G)–(L). Panel (M) shows PCR results for GATA4 in OFCs and FFCs in passage 2.

**Figure 4:**
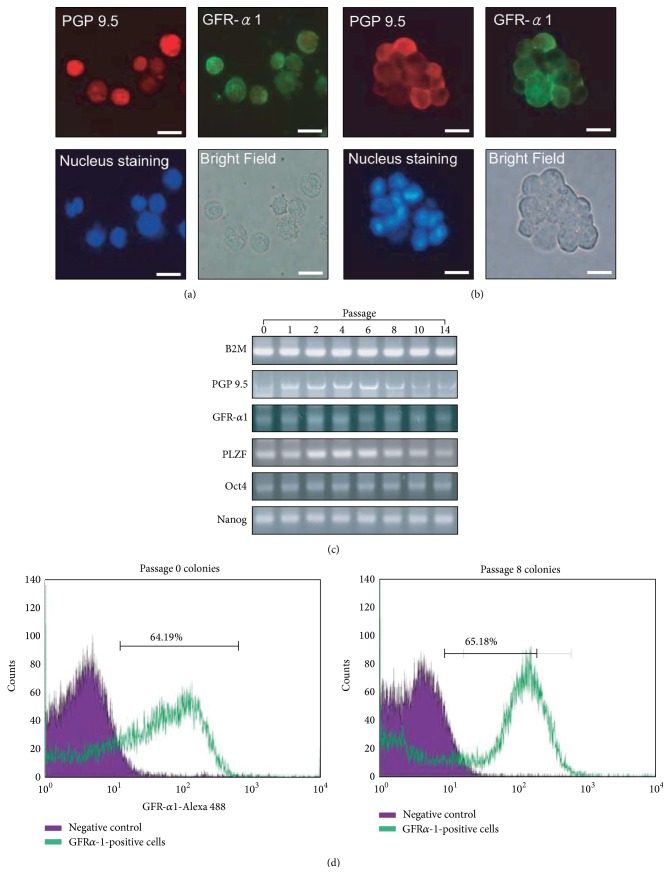
Characterization of germ cell-derived colonies in subculture. The GDC cells in passages 2 and 11 were stained with antibodies against PGP 9.5 and GFR*α*-1 ((a) and (b)), while the nuclei were stained with DAPI. Expression of germ and stem cell markers is shown in panel (c). The proportion of GFR*α*-1-positive cells, analyzed in passages 0 and 8, is shown in panel (d). Scale bars indicate 10 *μ*m in panels of (a) and (b).

**Figure 5:**
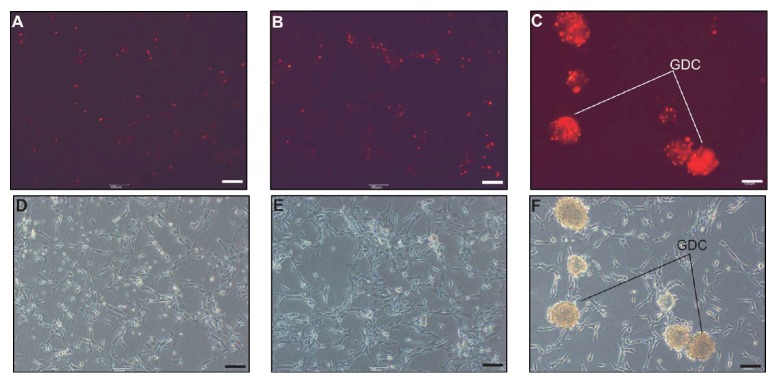
Subculture of frozen germ cell-derived colonies. The cells from frozen colonies, which were labeled with red trackers in passage 13, were maintained in the colonies of passage 14 ((A), (B), and (C)). Panels (D), (E), and (F) show the bright-field images of panels (A), (B), and (C), respectively. Scale bars indicate 100 *μ*m in all the panels. GDC: germ cell-derived colony.

Red-labeled GDC cells, from passages 1 and 10, were cultured with FFCs to confirm that GDCs can be maintained in subculture. Cells subcultured with GDC cells in passage 1 were mostly red in the colonies in passage 2, and the morphology of the GDCs was similar to those in Figures 2(J)-2(K) (Figures 3(A) and 3(B)). Similar observations were made in passage 11, when cells from colonies in passage 6 were subcultured with FFCs (Figures 3(C) and 3(D)). In neonatal testis tissues, GATA4, which is involved in mammalian testis development, was detected in the Sertoli and interstitial cells, but not in testicular germ cells that were stained with PGP 9.5 (Figures 3(E) and 3(F)). Further, immunocytochemistry of TTCs revealed that testicular germ cells were positive for PGP 9.5 antibody and GATA4 (Figure 3(G)). PGP 9.5- and GATA4-negative cells were also detected in the TTCs (Figure 3(G)). Nuclei of the cells in Figure 3(G) were stained with DAPI (Figure 3(H)). Bright-field microscopic examination revealed the germ cells to be rounded, measuring around 10 *μ*m in diameter, while the testicular somatic cells appeared to have irregular forms (Figure 3(I)). In addition, immunocytochemistry was performed for feeder cells that were attached to the well on day 10 at passage 2. No protein was detected in the negative controls (Figure 3(J)). Among OFCs, both GATA4-positive and GATA4-negative nuclei were detected (Figure 3(K)). In contrast, all attached FFCs were GATA4 positive (Figure 3(L)).* GATA4* transcript levels were also higher in the attached cells in FFCs in passage 2 than in passage 2 OFCs (Figure 3(M)). To investigate the characteristics of the colonies in each passage, immunocytochemistry, RT-PCR, and flow cytometry were performed. PGP 9.5 and GFR*α*-1 were detected in GDC cells collected in passages 2 and 11. Both the nuclei and morphology of these cells were normal (Figures 4(a) and 4(b)). Further, our RT-PCR results showed that the germ cell markers* PGP 9.5*,* GFRα-1*, and* PLZF* were expressed in the GDCs in passages 0 through 14; GDCs cultured with FFCs from frozen GDC cells at passage 13 had the same characteristics as those cultured from GDC cells at passages 0 to 10. Furthermore, GDCs at all the passages were found to express the stem cell markers* Oct4* and* Nanog* (Figure 4(c)). Our indirect flow cytometry analyses revealed 63.2 ± 4.51% and 67.31 ± 6.73% GFR*α*-1-positive GDC cells at passages 0 and 8, respectively (Figure 4(d)); GFR*α*-1-negative cells were also observed, but these were considered somatic cell contamination that occurred during GDC collection. To confirm long-term maintenance, cells labeled red from the frozen GDCs at passage 13 were subcultured with FFCs. On day 2, the feeder cells were attached to the bottom of the wells, while the labeled cells were evenly distributed (Figures 5(A) and 5(D)). On day 5, the labeled cells were attached to the feeder cells in the well (Figures 5(B) and 5(E)). On day 10, the labeled cells were found clustered on the feeder cells (Figures 5(C) and 5(F)).

## 4. Discussion

Several groups have reported the culture of SSCs of domestic animals. Aponte et al. reported that the StemPro-34 medium with 4 growth factors was optimal for short-term culture (15 days) of bovine SSCs. They further expanded the culture period up to 33 days, and when the cells cultured for 30 days were transplanted into the seminiferous tubules of nude mice, proliferation of type A spermatogonia was observed [9]. In pigs, short-term culture of gonocytes has been reported. Goel et al. cultured purified gonocytes in DMEM/F12 supplemented with 10% FBS in the absence of specific growth factors for 7 days. They observed cell proliferation and subsequent formation of colonies [10]. Further, Kuijk et al. reported that cell lines obtained from neonatal pig testes and cultured in the StemPro-34 medium could be maintained for up to 9 passages after cessation of proliferation and that growth factors affect the culturing of porcine spermatogonia [11]. However, the lack of established feeder cells limits the long-term culture of SSCs from domestic animals as used in mice even though growth factors have an important role in short-term SSC culture. Previously, we found that a temperature of 31°C, which positively correlates with cell growth, is optimal for GDC formation. In addition, GDCs were not detected in the presence of mitomycin-C-treated feeder cells (like MEFs and STO cells) [7]. In this study, we purified somatic cells from TTCs using density gradient separation and showed that the purified FFCs can be used for subculture of porcine GDCs. The GDCs were maintained in each passage as cell lines despite a few germ cells being present in the isolated somatic cells.

PGP 9.5 is known to be specifically expressed in porcine spermatogonia* in vivo* [12] and is often used as a marker for the same [7, 10]. GFR*α*-1, which combines with GDNF, is a cell surface receptor of undifferentiated spermatogonia in rodents, and it is often used to sort undifferentiated spermatogonia [13–15]. In previous studies, we found that* PGP 9.5* and* GFRα-1* transcripts are highly expressed in porcine GDCs and spermatogonia, respectively [7, 8]. Recent studies have shown that these markers are constitutively expressed in colonies of all passages. It has been reported that Nanog, Oct4, and Sox2, often used as stem cell markers, function cooperatively in the regulatory network of self-renewal and pluripotency [16]. Expression of* POU5F1* and* NANOG* transcripts has also been reported in domestic animals. For instance, higher mRNA expression of both* POU5F1* and* NANOG* was detected in porcine colonies [7]. Further,* POU5F1*, but not* NANOG*, was expressed in mouse germinal stem cells cultured under serum- and feeder-free conditions [17] and in porcine germ cell colonies [11].* POU5F1* transcripts were detected in 20-week-old testes but not in neonatal ones (2–7 days) [18]. In our previous results,* POU5F1* and* NANOG* mRNAs were highly expressed in GDCs cultured in the StemPro-34 medium at 31°C, and GFR*α*-1-positive cells expressed* Oct4* and* Nanog* mRNA [7, 8]. Based on these reports, it seems that germ and stem cell markers are expressed in cultured germ cells of domestic animals. In this study, cells of the colonies at each passage were found to be positive for GFR*α*-1 and PGP 9.5, as well as for stem cell markers, in RT-PCR, immunocytochemistry, and flow cytometry analyses. These results suggest that the GDCs stably maintain germ and stem cell characteristics during subculture.

GATA4 is known to be involved in the development and function of the mammalian testis [19]. In humans, the p.Gly221Arg mutation in GATA4 leads to anomalous testicular development [20]. GATA4 is also a key regulator of Sertoli cell function in adult mice [21]. It plays an integral role in the development of testicular steroidogenic cells [22]. Furthermore, GATA4 was also detected in the nuclei of mouse and human granulosa and thecal cells [23, 24]. In addition, the nuclei of 90–95% granulosa cells of porcine primordial, unilaminar, multilaminar, and antral follicles of different sizes stain positive for GATA4 [25]. McCoard et al. reported that GATA4 localizes to the coelomic epithelium of gonads and to the Sertoli and follicle cells before and after sex differentiation, respectively [26]. It is clear from these reports that GATA4 plays an important role in gonadal somatic cells that are involved in spermatogenesis and oogenesis. In this study, we found GATA4-positive cells in TTCs and testis tissues. FFCs cultured with the colonies, which were attached to the culture dish, were positive for GATA4. Moreover, GATA4 expression was stronger in FFCs than in OFCs, and it was detected in Sertoli cells of neonatal pig testes. These results suggest that GATA4-positive cells support GDC formation in two-dimensional culture and that, during culture with GDCs, the GATA4-expressing FFCs probably play a role similar to that of somatic cells in the testes—supporting germ cell growth and proliferation.

## 5. Conclusions

In conclusion, isolated GATA4-positive somatic cells can be used as feeder cells for long-term culture of porcine GDCs (>4 months) and the GDCs thus cultured maintain germ and stem cell characteristics during subculture.

## Figures and Tables

**Table 1:** Number of cells collected from colonies in subculture with fresh feeder cells.

Passage	Number of collected cells from colonies	Number of used cells for subculture
0	1.95 ± 0.03 × 10^6^/12 wells	2 × 10^4^/well
1	6.6 ± 0.9 × 10^5^/12 wells	2 × 10^4^/well
2	5.9 ± 0.6 × 10^5^/12 wells	2 × 10^4^/well
3	8.7 ± 0.9 × 10^5^/12 wells	2 × 10^4^/well
4	1.14 ± 0.12 × 10^6^/12 wells	2 × 10^4^/well
5	1.12 ± 0.11 × 10^6^/12 wells	2 × 10^4^/well
6	1.01 ± 0.26 × 10^6^/12 wells	2 × 10^4^/well
7	1.06 ± 0.12 × 10^6^/12 wells	2 × 10^4^/well
8	1.61 ± 0.36 × 10^6^/12 wells	2 × 10^4^/well
9	1.25 ± 0.2 × 10^6^/12 wells	2 × 10^4^/well
10	1.31 ± 0.58 × 10^6^/12 wells	2 × 10^4^/well
